# Needs and Experiences in Psychiatric Treatment (NEPT)- Piloting a Collaboratively Generated, Initial Research Tool to Evaluate Cross-Sectoral Mental Health Services

**DOI:** 10.3389/fpsyt.2022.781726

**Published:** 2022-01-27

**Authors:** Sebastian von Peter, Helene Krispin, Rosa Kato Glück, Jenny Ziegenhagen, Lena Göppert, Patrick Jänchen, Christine Schmid, Anne Neumann, Fabian Baum, Bettina Soltmann, Martin Heinze, Julian Schwarz, Timo Beeker, Yuriy Ignatyev

**Affiliations:** ^1^Department of Psychiatry and Psychotherapy, Brandenburg Medical School Theodor Fontane, Immanuel Klinik Rüdersdorf, Rüdersdorf, Germany; ^2^ExPEERienced- Experience With Mental Health Crises- Registered Non-profit Organization, Berlin, Germany; ^3^Zentrum für Evidenzbasierte Gesundheitsversorgung, Technische Universität Dresden, Dresden, Germany

**Keywords:** PREM, peer research, coproduction, collaboration, tokenism, experience, user involvement

## Abstract

**Background:**

Research tools to evaluate institutions or interventions in the field of mental health have rarely been constructed by researchers with personal experience of using the mental health system (“experiential expertise”). This paper presents a preliminary tool that has been developed within a participatory-collaborative process evaluation as part of a controlled, multi-center, prospective cohort study (PsychCare) to evaluate psychiatric flexible and integrative treatment, FIT for short, models in Germany.

**Method:**

The collaborative research team consisting of researchers with and without experiential expertise developed 12 experiential program components of FIT models by an iterative research process based on the Grounded Theory Methodology. These components were transformed into a preliminary research tool that was evaluated by a participatory expert panel, and during a pilot and validation study, the latter using a random sample of 327 users from 14 mental health departments. Internal consistency of the tool was tested using Cronbach's alpha. Construct validity was evaluated using a Principal Components Analysis (PCA) and a Jonckheere Terpstra test in relation to different implementation levels of the FIT model. Concurrent validity was tested against a German version of the Client Satisfaction Questionnaire (ZUF-8) using correlation analysis and a linear regression model.

**Results:**

The evaluation of the expert panel reduced 29 initial items to 16 that were further reduced to 11 items during the pilot study, resulting into a research tool (Needs and Experiences in Psychiatric Treatment—NEPT) that demonstrated good internal consistency (Cronbach's alpha of 0.89). PCA yielded a 1-component structure, which accounted for 49% of the total variance supporting the unidimensional structure of the tool. The total NEPT score increased alongside the increasing implementation of the FIT model (*p* < 0.05). There was evidence (*p* < 0.001) for convergent validity assessed against the ZUF-8 as criterion measure.

**Conclusions:**

The NEPT tool seems to be promising for further development to assess the experiences with and fulfillment of needs of psychiatric care models from the perspective of users. This paper demonstrates that it is possible to use a participatory-collaborative approach within the methodologically rigorous confines of a prospective, controlled research design.

## Introduction

Research tools and psychometric scales used to evaluate institutions and interventions in the field of mental health have mostly been constructed by clinical scientists with no personal experience of the psychiatric care system, mental crises and disabilities or recovery from them (in the following designated as “experiential expertise”). Yet, there is a rich tradition of research and knowledge production by scholars with experiential expertise that has been contributing to the mental health field for more than two decades in various countries (1–4). Using different epistemological and theoretical approaches (5–8), these studies frequently articulate valid criticism toward the current medicalized approach to psychiatric care (2), the psy-centrism of contemporary social infrastructures (8), as well as the appropriation of contrasting perspectives and positions (1), resulting into the silencing of possible alternatives—also on the level of knowledge production.

Given this context, only a few research groups in the field of mental health, led by or including researchers with experiential expertise, have been able to establish. One of these exceptions is SURE (Service User Research Enterprise)/United Kingdom, hosting exclusively researchers with experiential expertise who investigate and evaluate various health care services using self-developed criteria, standards, and instruments (9). Developed by this group and others, several scales have been created by researchers with experiential expertise: As early as 1996, Diana Rose's hybrid team created the “CONTINU-UM scale” to evaluate the continuity of psychiatric treatment (10). In the following year, Rogers created the “Empowerment Scale,” using expertise from a participatory board staffed by activists from self-help groups (11). The “Evans VOICE Inpatient Care Scale” was also developed in a participatory way and surveys the aspects of care that users consider to be important (12). The questionnaire about the process of recovery (QPR) was developed by a collaborative research team and may assist users to set treatment goas (13). The last example is the “CEO-MHS,” for which researchers with experiential expertise created a questionnaire to record user satisfaction (14).

This paper presents the first steps of developing a novel research tool that aims at evaluating the experiences and fulfillment of needs during psychiatric treatment from the perspective of users. This tool was developed during a participatory-collaborative process evaluation as part of a controlled, multi-center, prospective cohort study (PsychCare), aiming at evaluating psychiatric, innovative, flexible, and integrative treatment (FIT) models in Germany (15). These FIT model projects are mainly hospital-based and enable a more need-adapted, cross-sectoral service delivery, including complex outpatient forms of psychiatric treatment (16). Our approach involved the continuous collaboration between researchers with and without experiential expertise with the psychiatric care system, crises and disabilities or recovery from them (17). It is based on a cooperation that neither intends to meet the strict and egalitarian criteria of co-production (18, 19), nor the systematic involvement of actors in the field under investigation, as practiced in participatory research projects (20). Instead, our mode of collaboration allowed to substantially build upon knowledge of researchers with experiential expertise within the methodologically rather rigorous confines of a prospective, controlled cohort study.

The overall aim of the PsychCare study was to examine the benefits, costs, and efficiency of more flexible, continuous, and setting-integrated treatment models in Germany in comparison to standard care currently provided. Following the MRC Guidelines for the Evaluation of Complex Evaluations (21), one part of this study included a participatory process evaluation that was realized by the mentioned collaborative teamwork. The main results of this process evaluation will be presented elsewhere (22). This paper focusses on the collaborative development of a research tool during this process evaluation that aimed at evaluating the experiences and fulfillment of needs during psychiatric treatment from the perspective of users. The construction of this research tool and the initial steps of piloting and validation will be described, followed by a discussion on its value within the context of this study and beyond.

## Materials and Methods

The PsychCare study is financed by the German Innovation Fund of the Federal Joint Committee (G-BA) (grant reference no. 01VSF16053), which invests resources from the health care insurance system in researching innovative health programs (23). The study is aimed at evaluating innovative psychiatric treatment models that have gradually been developed following the 2012 introduction of the § 64b of the German Social Code Book V (22). Results of previous studies on this topic are published elsewhere (16, 21, 22, 24–29). The above-mentioned law enables the implementation of more flexible and integrative, psychiatric treatment models (FIT models) based on a Global Treatment Budget (GTB). Given the rather rigid and fragmented nature of the German health care system, these FIT models allow for more user-oriented and outpatient forms of treatments (30). As a result, users stay mainly in their home environment but can also be treated flexibly in the clinic with less bureaucratic hurdles. Ideally, this allows better integration of the treatment into the user's everyday life and a better insight into their reality of life by the staff (31).

The aforementioned GTB targets a fixed number of people to be treated per year. How this budget is used, for which treatment, in which settings and for what purpose is decided by the relevant institution. A total of 22 of these FIT models can currently be found in the hospital sector in Germany.

### Team Structure and Cooperation

The results presented in this manuscript build on a previous study, Eva-Mod64 (22), in which 13 FIT hospital departments were evaluated between 2016 and 2017, resulting into the development of 11 process and structure-related program components of FIT models (16, 22, 24). Whereas this precursor study was carried out only by researchers without experiential expertise, the team of the PsychCare participatory process evaluation was staffed by both researchers with (in the following “experiential experts,” EE) and without experiential expertise (”conventional researchers,” CR). This team composition was chosen to direct the evaluative focus on the specific experiences of FIT model users. The three EE involved in the team were researchers with and without academic degrees. The CR group consisted of two medical students, two paid researchers and the team principal, the latter working in psychiatry but not having personal experiences as mental health service users.

The team met as a whole or in subgroups (CR only, EE only, or EE + CR). In between meetings, the team members worked individually, alone or in tandems, consisting of one EE and one CR each. In addition, supervision sessions took place three times per year, covering the whole group or CR and EE as individual groups. During these supervisory sessions, the collaborative approach and its impact on the research results were reflected upon. The results of this work will be published elsewhere (32). The whole team contributed to all phases of this project, and also as authors of this paper.

### Construction of the NEPT Research Tool

**Figure 1:**
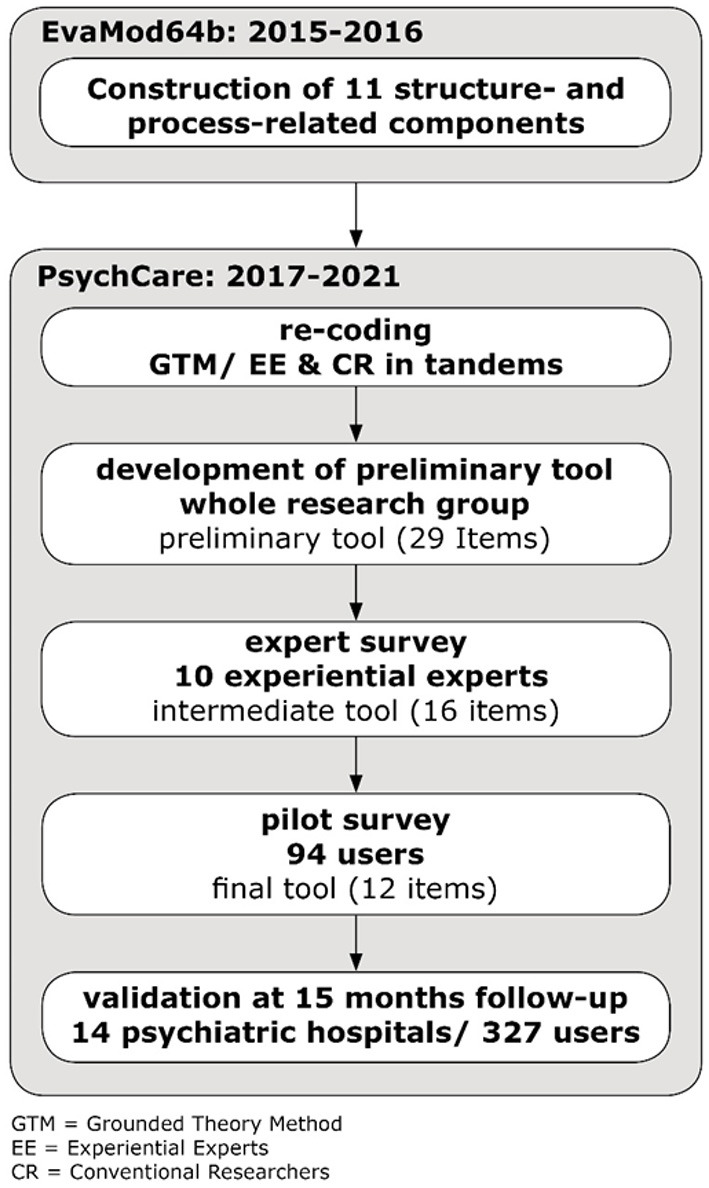
Multi-step, collaborative construction of the NEPT research tool.

The construction of the NEPT research tool was carried out in several steps shown in Figure 1 to reduce complexity. Chapter 2.2 describes the construction of the experiential components and the preliminary items of the NEPT research tool. An ethics vote of the TU Dresden dated 07.09.2017 was available.

#### Construction of the Experiential Components

At the beginning of the study, the 15 core transcripts containing focus group material from the EvaMod64b precursor study (25) were re-coded to familiarize the team with the research topic. An evaluation method based on Grounded Theory Methodology (GTM) (33) was chosen, as the GTM allows for the systematic inclusion of various positions and forms of knowledge during a coding process, a high degree of process orientation and flexibility, and the systematic handling of conflicting perspectives and irritations (31). The coding of these transcripts was carried out in tandems of EE and CR using a computer assisted qualitative data analysis software *NVivo* (34) and the 11 process- and structure-related components of the precursor study as deductive categories (26). While the CR coded deductively, the EE were encouraged to add open codes, which enabled them to systematically feed personal experiences and/or collectivized forms of experiential knowledge into the coding process.

**Figure 2:**
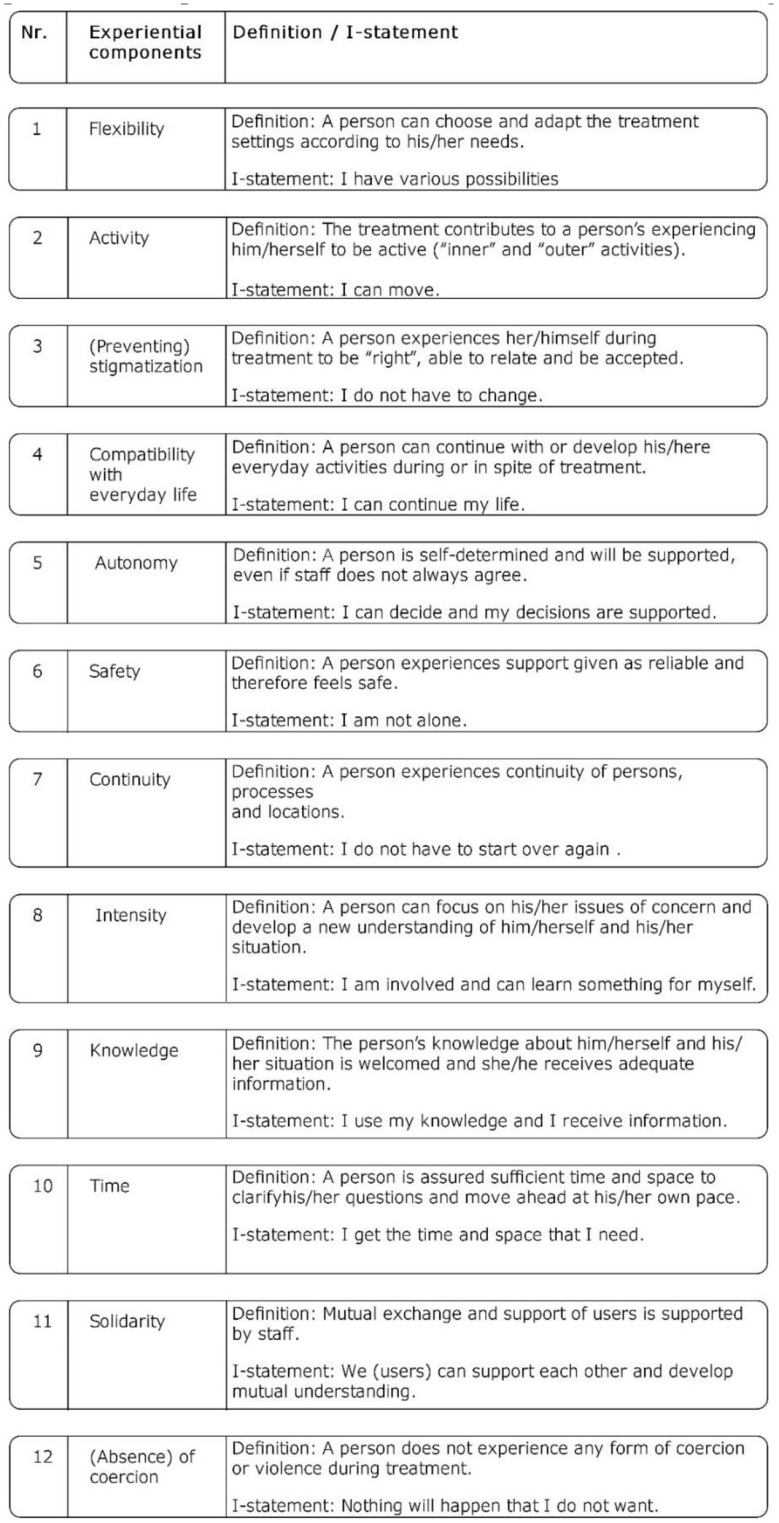
The developed 12 experiential components that reflect the experiences and fulfillment of needs of psychiatric treatment from the perspective of service users. To this end, they were framed as I-statements and their definitions were given accordingly.

This process enabled “creative chaos” (30) allowing the group to discover and code new aspects and to open-up the possibility of systematically enriching the insights from the precursor study through experiential expertise and generalizing them further (22). As a result, a set of 12 so-called experiential program components emerged (Figure 2), aiming at capturing the experience of the FIT model users. As these components emerged from the coding process described above and the underlying experiential knowledge of the EE involved, they were framed as “I-sentences” to highlight their experiential character. They were further defined, repeatedly discussed, and finally agreed upon by the whole group and, in accordance with GTM, systematically linked to each other, as well as to the process and structure-related components of the precursor study.

#### Construction of the Questionnaire Items

The experiential components served as a theoretical basis to develop the research guidelines for the qualitative part of the process evaluation (35). They were further used to construct items of a standardized research tool that assesses the experiences with and fulfillment of needs of the evaluated care models from the perspective of users. This tool was introduced during the 15 months follow-up of the PsychCare study's outcome evaluation to assess the experiences of a larger number of users in a more standardized way (15), to be able to triangulate the results of the study's process with those of the outcome evaluation. A second aim was to better understand the value of an evaluative construct to assess user experiences, and in how far such construct may share similarities with other constructs, for example to evaluate treatment satisfaction. Literature on this question usually targets PREM constructs (patient reported experiential measures) (36, 37), however generally not involving experiential expertise during their processes of construction.

To this end, the experiential components were transformed in several stages into questionnaire items that ultimately resulted in a questionnaire called “Needs and Experiences in Psychiatric Treatment” (NEPT). The first stage consisted in converting the I-sentences of the components into questions by the EE subgroup. These questions were discussed and further developed into 2 or 3 different questions per item by the whole group. The component “*Flexibility*” (see Figure 2), for instance, was assigned to the questions: “Were you treated overall in the settings that were suitable for you (full-time inpatient, day clinic, at home)” and “Did the change between the settings take place in a way that was suitable for you?.” To facilitate understanding and to do justice to their experiential nature, all questions selected were then re-converted into I-statements that, finally, were endued with a five-level Likert scale (strongly disagree, disagree, neither agree nor disagree, agree, strongly agree).

### Piloting the NEPT Research Tool

After developing the questionnaire items (s. 2.2), they were piloted and validated. This took place in three surveys, first in an expert survey, then in a pilot survey and finally using a larger population during the 15 months follow-up of the PsychCare study's outcome evaluation.

#### Expert Survey

The content validity of the questionnaire items was calculated using an expert panel (38). The expert survey targeted a group of 10 EEs, also deviating from the precursor study, in which only CR had undertaken this phase of the validation process (21). For this expert survey, the group consisted of users, mental health activists, patient representatives, recovery companions and peer and user researchers, with the majority of these experts having several of these identities. Overall, women predominated in the group (7:3), members ranged in age from 26 to 72 years.

The preliminary items were presented in two rounds to the expert group that was asked to assess which of the assigned questions best captured the essence of the underlying components. A rating from 1 to 3 was given, 1 standing for ”essential,” 2 for “appropriate but not essential” and 3 for “not essential.” At least half of the experts had to agree that a question should be classified as ”essential“ to confirm its content validity (3). Based on the results of this expert survey, a scale with five levels of ”not at all applicable,” “somewhat inapplicable ,” ”neutral,” “somewhat applicable ” and “fully applicable” was assigned, which served to evaluate the items during the pilot survey.

Based on the results of the expert assessment, the final questionnaire was developed, which contained a total of 16 items, assigned to the 12 experiential components on which it was based.

#### Pilot Survey

The pilot survey included 94 users of one of the FIT model departments that was not included in the main study, with the sample drawn from three treatment settings (outpatient clinic, day clinic and hospital ward) according to a quota principle. Respondents were asked to rate the items using the above-mentioned five-point scale. In addition, the respondents' detailed comments on individual items were recorded.

Based on the feedback of the participants, the correlations and reliability of the items were determined. With reference to previous studies (39), in which socially desirable response behavior was shown to occur in the evaluation of health care services, the items were coded as follows: “not at all applicable” to “neutral” = 0, ”somewhat applicable“ = 1 and ”fully applicable“ = 2. Further, selection of items on the questionnaire was based on the principle of excluding items with low internal consistency with the scale, the cut off for dripping items being set at ≤ 0.7. This strategy, called alpha maximization (38), was used with the greatest caution, as it can lead to the elimination of items with low selectivity, necessary to distinguish all areas of the dimensional spectrum. In addition, this strategy can lead to a reduction of content validity, which is why the research team also took content considerations into account when selecting the items (40).

#### Validation of the NEPT Research Tool

The developed NEPT questionnaire was handed out to the investigated users of the 15 months follow-up of the PsychCare outcome evaluation. For details on inclusion and exclusion criteria see Soltmann et al., (15). Socio-demographic characteristics of the sample were assessed using descriptive statistics.

##### Testing Internal Consistency

The internal consistency of the NEPT tool was assessed by estimating item–total correlations by Spearman's rank, which expresses the degree to which the items of an instrument are measuring the same attribute. Additionally, the correlation matrix was checked. The size of the correlations was based on the following interpretation limits according to Cohen (41): 0.10 > *r* < 0.30, small effect size; 0.30 > *r* < 0.50, medium effect size and *r* > 0.50, large effect size. Internal consistency was also estimated using a Cronbach's alpha reliability coefficient. A Cronbach's α > 0.6 and ≤ 0.7 was considered an acceptable value; a value >0.7 and <0.9 a good value; and a value of 0.9 or higher indicated excellent reliability (42). For the pilot testing, alpha maximization was used as a criterion for item elimination; the cut off for dropping items was 0.7.

##### Testing Validity

Validity was assessed in several ways: First, an exploratory Principal Components Analysis (PCA) was conducted to evaluate the underlying structure of the instrument (43). To test for the adequacy of PCA, we used the Kaiser-Meyer-Olkin measure (should be ≥ 0.5) (ibid) and the Bartlett test of sphericity (ibid) (should be significant). A strict cut-off of factor loading of > 0.50, used by other researchers (44) was adopted. This method is primarily used to explore covariation without having a prior hypothesis or theory (45). In our case, the number of components to extract were based on three criteria: the Eigenvalue >1 (Kaiser), the Velicer MAP criterion (Polychoric correlations), and the Velicer MAP criterion/ 4th power (46, 47) using simulated polychoric correlation matrices.

In a second step and to support construct validity, known-groups validity was examined, testing hypothesized groupings of the survey outcomes, and detecting differences between them (48). A linear trend was tested across participants from three mental health hospital groups with different health care providing levels (centers providing standard health care, centers providing both FIT and standard treatment, and centers exclusively providing FIT treatment), using the Jonckheere-Terpstra test, one-tailed from a Monte Carlo simulation (10 000 samples) (49). The hypothesis was that these groups were ordered in a specific sequence, expecting that the participants from the hospital groups with a higher level of the FIT treatment would have higher NEPT total scores.

Third, concurrent validity was assessed by comparing the total NEPT scores with the total ZUF-8 scores (50), a German version of the Client Satisfaction Questionnaire CSQ-8 (51) that was also used at the 15 months follow-up of the PsychCare study. Concurrent validity was analyzed both by calculating a Pearson's correlation (38) and by using a multiple linear regression model, adjusted for the influence of the two demographic covariates gender and age. Missing data of NEPT and ZUF-8 questionnaires were not imputed. The size of the Coefficient of determination (R^2^) was based on the following interpretation limits according to Cohen: R^2^ <0.02—very weak, 0.02 ≤ R^2^ <0.13—weak, 0.13 ≤ R^2^ <0.26—moderate, R^2^ ≥ 0.26—substantial (52). We expected to find a significant correlation with a large effect size between ZUF-8 total and the NEPT total-scores using correlation analysis and a significant association with a substantial R^2^ in a linear regression analysis with the NEPT total value as a dependent and the ZUF-8 total as an independent variable.

The significance level was set at *p* ≤ 0.05. Most analyses were performed with the Statistical Package for Social Sciences (SPSS), version 23.0 for Windows. The Velicer MAP criterion and the Velicer MAP criterion/4th power were examined with the *r* package “random.polychor.pa” running in *r* version 4.0.5 (45).

## Results

### Cooperation Within the Group

A detailed description of the teams' collaborating processes and experiences while conducting this study has been published elsewhere (32). As described above, staffing the team with a mix of researchers with and without experiential expertise, organizing our work in different sub-groups and tandems, made it possible to systematically incorporate experiential expertise throughout the whole research process. It opened-up an “area of negotiating meaning and representation” (53), enabling new forms of knowledge and the recombination of different forms of knowledge to evaluate and (hopefully) ultimately improve psychiatric treatment.

At the same time, the research group was located within a privileged site of knowledge production (university) and entrenched within the confines of a rather traditional research design (prospective, controlled study). Thus, collaborative knowledge production was subject to various contingencies, emerging from academic rules and parameters that also defined to a certain extent the roles and responsibilities of the researchers involved. This led to a rather disciplined form of experiential expertise coming into play, that stretched the standard criteria of health service research and/or psychiatric discourse but ended up subjugating its emancipatory potential to the authority of scientific knowledge and academic knowledge production. Longstanding, structural inequalities of university knowledge production as well as rather strict (mental) health service research epistemologies remained largely untouched, leading to various frustrations especially on the side of the researchers with experiential expertise [for further details see Beeker et al. (32)].

### Construction of the NEPT Research Tool

#### Construction of the Experiential Components

Over the course of the construction process, 12 experiential components were developed based primarily on the knowledge of the researchers with experiential expertise. The differences between these experiential components and the set of process and structure-related components from the precursor project, and the role that experiential knowledge played in producing them, will be described elsewhere (22). At this point, it is sufficient to point out that the collaboration between researchers with and without experiential expertise resulted in (1) a number of new components with new areas of content, (2) the re-definition and/or -operationalization of the previous components, in some cases considerably, and (3) further generalization of these experiential components, transcending their original evaluative focus on FIT models to move toward the evaluation of ”good psychiatric care“ (see Discussion Section). A compilation of the 12 experiential components and their definitions can be found in Figure 2.

#### Construction of Questionnaire Items

A total of 29 survey items were developed in several steps, with 2-3 items assigned to each of the experiential components. The items were listed and can be found in the accessory material to this manuscript (Supplementary Table A1).

### Piloting of the NEPT Research Tool

#### Results of the Expert Survey

In the expert survey, 16 out of the 29 survey items were rated “substantial” by at least half of the experts. Thereafter, the following number of items remained in the preliminary questionnaire: *Flexibility* = 1 item, *Activity* = 1 item, *Avoidance of stigmatization* = 1 item, *Compatibility* = 1 item, *Autonomy* = 2 items, *Safety* = 1 item, *Continuity* = 2 items, *Intensity* = 1 item, *Knowledge* = 2 items, *Time* = 1 item, *Solidarity* = 1 item, *Absence of coercion* = 2 items.

The remaining 13 items were eliminated. The main reasons for the low rating of eliminated items were that, according to experts, they did not sufficiently reflect the essential aspects of experience or were redundant, such as the items: “Switching between different settings went so well that it suited me” (eliminated due to redundancy), “I was supported in developing activities that were helpful to me” (eliminated as activity was not sufficiently specified), “The treatment conditions (behavior of personals, rooms, regulations) allowed me to look at myself benevolently” (elimination as it does not sufficiently differentiates between self-stigmatization and stigmatization from outside), “During my treatment I was supported in developing skills that I can also use in my life” (eliminated as the “life” was too unspecific), “I experienced support and safety during the treatment” (eliminated as it mixes two items), “During my treatment I was able to deal with my own situation” (eliminated as it was too vague), “I was given sufficient time during the treatment” (eliminated due to redundancy), “The team encouraged users to support one another” (eliminated as it does not thematize exchange between the users).

#### Results of the Pilot Survey

Using the alpha maximization method, nine of the remaining 16 items were found to have relatively low discriminatory power. Considerations of the research team led to the retention of four items relating to the characteristics of *Compatibility with everyday life, Safety, Time, Solidarity*, and the elimination of five items relating to the characteristics of *Autonomy* (1 item), *Continuity* (1 item), *Knowledge* (1 item), and- unfortunately- *Avoidance of coercion* (2 items). The items relating to the last characteristic were eliminated due to comments of the respondents which clearly indicated they had difficulties answering the corresponding questions. The final version of the scale contained 11 items (**Table 2**), one item each for *Flexibility, Activity, Avoidance of stigmatization, Compatibility with everyday life, Autonomy, Safety, Continuity, Intensity, Knowledge, Time, Solidarity*. The Cronbach's alpha value for the overall scale was 0.82 (0.77–0.88).

#### Validation of the NEPT Research Tool

A sample of 374 participants was tested during the 15 months follow-up of the PsychCare study. Because of missing data, 47 cases were excluded from further analyzes. The final sample included 327 people who were treated in 14 mental health centers, including 140 male and 187 female participants that were part of the study. The mean age was 47 (±13.48) years for the men, and 47.9 (±13.94) years for the women. Table 1 shows the mean scores of the NEPT items for both genders. The mean total NEPT score for the entire sample was 4.02 (±1.19). Women [M (SD) = 4.06 (0.71)] had a slightly higher total score than men [M (SD) = 3.95 (0.68)].

**Table 1:** Mean scores of NEPT items and total score.

**Item**	**Men (*****N*** **=** **140)**		**Women (*****N*** **=** **187)**		**Full sample (*****N*** **=** **327)**	
	**M**	**SD**	**M**	**SD**	**M**	**SD**
Flexibility	3.96	1.21	4.06	1.18	4.02	1.19
Activity	3.97	0.99	3.89	1.1	3.93	1.05
Preventing (stigmatization)	4.3	0.8	4.20	1.04	4.24	0.94
Compatibility with everyday life	4.03	1.05	4.01	1.16	4.02	1.11
Autonomy	3.9	1.02	4.09	0.91	4.01	0.96
Safety	4.16	0.89	4.26	0.84	4.22	0.86
Continuity	4.02	1.01	4.22	0.87	4.14	0.93
Intensity	3.92	0.98	4.12	0.95	4.04	0.96
Knowledge	3.97	1.04	4.07	0.99	3.95	1.02
Time	3.62	1.08	3.80	1.05	3.72	1.07
Solidarity	3.81	1.04	3.96	1.03	3.9	1.04
Total score	3.95	0.68	4.06	0.71	4.02	0.70

##### Internal Consistency

Table 2 shows the inter-correlations between the remaining 11 items as well as correlations between the items and the NEPT total score. All correlations were significant at the level not <*p* < 0.01 except for the correlation between the Items *Compatibility with everyday life* and *Solidarity*. Except for these two items, the coefficients ranging from 0.54 to 0.77 were calculated for the corrected item-total correlations, which indicated adequate homogeneity of items. The correlations of the items *Compatibility with everyday life* and *Solidarity* were 0.45 and 0.44, respectively, which indicated that these items contributed relatively less to the tool. According to the inter-item correlation matrix, no items were above 0.80, indicating a lack of multicollinearity (41). The Cronbach's alpha coefficient for the summary scale was good (0.89). The Cronbach's alpha coefficient if item deleted ranged from 0.87 to 0.89, indicating that no items were unreliable. However, the contribution of the items *Compatibility with everyday life* and *Solidarity* for the internal consistency of the tool was critical.

**Table 2:** Correlations on NEPT items and Cronbach's alpha (α).

**Item**	**1**	**2**	**3**	**4**	**5**	**6**	**7**	**8**	**9**	**10**	**11**
1 Flexibility	1										
2 Activity	0.41	1									
3 Preventing (stigmatization)	0.53	0.52	1								
4 Compatibility with everyday life	0.38	0.31	0.36	1							
5 Autonomy	0.39	0.40	0.49	0.46	1						
6 Safety	0.46	0.35	0.55	0.35	0.52	1					
7 Continuity	0.37	0.23	0.38	0.36	0.45	0.45	1				
8 Intensity	0.44	0.45	0.58	0.35	0.48	0.57	0.43	1			
9 Knowledge	0.45	0.43	0.58	0.44	0.56	0.62	0.45	0.77	1		
10 Time	0.43	0.46	0.44	0.37	0.52	0.47	0.44	0.53	0.6	1	
11 Solidarity	0.20	0.33	0.34	0.11	0.27	0.40	0.25	0.41	0.38	0.42	1
Corrected item total scale correlation	0.56	0.54	0.69	0.45	0.63	0.71	0.55	0.73	0.77	0.67	0.44
Cronbach's α if item deleted	0.88	0.88	0.87	0.89	0.88	0.87	0.88	0.87	0.87	0.87	0.89

##### Validity

*Structural Validity.* Prior to performing the multivariate analysis, the adequacy of the correlation matrix of the scale was checked. The observed values KMO = 0.91 and Bartlett's Sphericity Test, χ^2^ = 1632.63, df = 55, *p* < 0.001 supported a multivariate analysis, which was carried out using PCA. Without fixing the number of components to extract, the PCA identified two components with Eigenvalue (Kaiser's criterion) >1 (5.37 and 1.03), conjointly accounting for 58.16% of the total variance: This solution clearly produced a general unipolar component, all items with positive loadings > 0.50, ranging from 0.53 (Item: *Compatibility with everyday life*) to 0.84 (Item: *Knowledge*). The second component aggregated items with lower component loadings (no items attained the component loading cut-off) and therefore was initially regarded as dubious. However, subsequent application of other criteria (Velicer MAP criterion and Velicer MAP criterion/4th power) confirmed the 1-component solution, which accounted for 48.65% of the total variances. Based on these results, the unidimensional structure of the tool was acknowledged.

The Jonckheere-Terpstra test results (z = 1.859, *p* = 0.03) showed that the NEPT total score differed based on the experiences in order (i.e., three independent groups: “centers providing standard health care, Mdn = 3.9,” “centers providing both FIT and standard treatment health care, Mdn = 4.0,” and “centers exclusively providing FIT treatment, Mdn = 4.1”) and therefore provided known-groups validity evidence for the scale.

**Figure 3:**
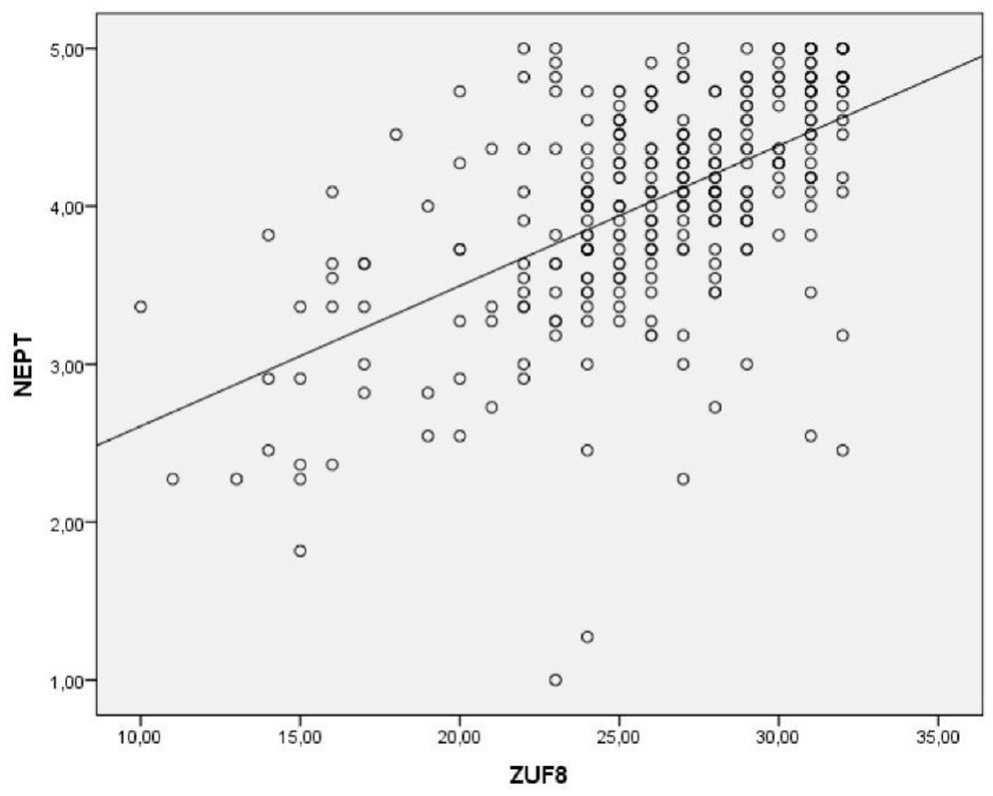
Scatterplot presenting the linear relationship between the ZUF-8 total score and NEPT total score. ZUF-8, German version of the Client Satisfaction Questionnaire CSQ-8; NEPT, Needs and Experiences in Psychiatric Treatment scale. Solid line represents a hyppthetical 1:1 relationship between total scores of both instruments.

*Concurrent Validity.* The Pearson's correlation analysis to assess the relationship between ZUF-8 total score and NEPT total score in a total of 299 participants preliminarily showed the relationship to be monotonic, as assessed by visual inspection of a scatterplot (see Figures 1, 3). As expected, there was a strong positive correlation between ZUF-8 total score and NEPT total score rs = 0.56, *p* < 0.001, indicating the tools are measuring comparable constructs. Using linear regression analysis, a significant association (*p* < 0.001) between total scores of both scales was found. The R^2^ for the overall model was 0.33 (adjusted R^2^ = 0.32), indicative for a substantial goodness-of-fit according to Cohen (41).

## Discussion

This paper presents the construction and validation of a collaboratively generated, preliminary research tool to evaluate the experiences and fulfillment of needs during psychiatric treatment from the perspective of service users. The instrument showed good internal consistency and its structural validity examination suggested its unidimensional structure. On known-group validity, a linear increasing trend of the total instrument score was observed across three independent mental health hospital groups as the level of flexible and integrative (FIT) psychiatric treatment increased. In addition, there was evidence for convergent validity assessed against the ZUF-8 as the criterion measure.

### “Experience” as a Construct

Our research tool operationalizes how psychiatric treatment was experienced by the users in relation to their needs. This focus fits the growing interest in assessing patients' experiences with health care services, meanwhile representing one of the three pillars in assessing the quality of health care services alongside clinical effectiveness and patient's safety (36, 37, 54). Online platforms for user input as well as internet-based reviews and ratings are increasingly developed to make room for critical feedback on the health care system and to give more space to the user experience (55). Quality assurance is increasingly focused on the user experience, with the aim of transforming care systems accordingly (56). And the users' experiences are playing an increasing role in research and evaluation, often justified on grounds of their intrinsic value, or findings that demonstrate associations between positive experiences and patient adherence, safety culture, and service utilization (37, 54).

Yet, a clear definition of what exactly counts as an experience is often lacking, most probably due to the complex nature of this construct (54). In the field of health service research, most confusingly, various notions are used interchangeably, such as user/patient-perspective, -reports, -perception, or -satisfaction Ahmed (36). In this manuscript and following Ahmed (36) and Price (54), experience refers to any users' perceptions of both objective facts and subjective evaluations (“Erleben” in German), also reflecting the evaluation of structures and processes that are not directly observable by them. In this sense, experience is an inherently multi-dimensional construct, encompassing needs, preferences, hopes, and expectations (57). It is deeply value-based at the same time opening-up potentials to serve as a useful proxy for assessing the quality of a received service.

As widely described, experience resembles the outcome parameter of patient satisfaction (37). This correspondence was confirmed by our results that demonstrated a convergent validity of both these constructs. Yet, the construct of ”experience“ was chosen, as it focusses more on concrete situations and is less reflexively charged (58). Further, as experiences are always intertwined with how they are evaluated, in our case with the question of what the treatment *was* or *should have been like* for the users, our construct also seems to relate to users' needs, as the extent to which a lack felt by the user had been eliminated through the services offered by the institution (59). Further, our construct generalizes experiences beyond those that merely relate to FIT departments, also building on users' experiences with the control group services. The FIT models having a broader scope in providing insights for the further development of the German mental health care system, our construct may be perceived to provide for a more general measure of ”good mental health care“ from the perspective of users- a hypothesis that will have to be confirmed in future studies.

Both experience- and satisfaction-based evaluation instruments are susceptible to subjective bias, being strongly linked to previous expectations, subjective judgment, social expectancy, and divergent perspectives (36, 37). In this context, more facts-based evaluation approaches are needed that increase objectivity in the evaluation of services, at the same time diminishing the possibilities for subjective interpretation. Examples in this context can be drawn from the development of fact-based PREMS and PROMS models (60, 61) that aim at evaluating key situations. A further development of our preliminary research tool in this direction, building on the qualitative findings of our participatory process evaluation (31), is planned as well as both its validation across various treatment models and care contexts.

### Impact of the Collaboration

Our work has shown that participatory-collaborative research undertaken together by CR and EE is indeed possible even within the confines of a rather conventional mental health research project. This collaboration was not free from academic, structural inequalities [see Section Cooperation Within the Group and (32)], at the same time opening-up space for the researchers with experiential expertise to contribute with their specific knowledge, leading to the development of the experiential components and the instrument based on them. In our view, the framework of a process evaluation, as it is recommended in the MRC Guidelines (21), is well suited to host such a form of systematic collaboration. In contrast, the design requirements related to other parts of the PsychCare study, e.g., using fixed outcome measures or analyzing routine data or health economic parameters, would have generated significantly less opportunities for such collaborative work. Thus, the largely inductive-qualitative logic of a process evaluation (62) seems to suit research involving a collaborative approach and may enable, as in our project, the step-by-step development of a collaboratively generated instrument to be used as an evaluative research tool.

As described, our collaborative work was built upon a previous non-collaborative evaluation of German FIT models during the EvaMode64b precursor project that- despite of the fact that it aimed at evaluating user experiences too- resulted into the development of a set of components useful to assess FIT specific processes and structures (25). Thus, the ongoing collaboration between researchers with and without experiential expertise within the PsychCare study enabled us to develop a research tool that is now more in line with the needs and experiences of service users, a finding that is also described in the literature: Opinions on what is and is not considered good care may differ largely, depending on whether users or practitioners have been questioned and by whom the related evaluative criteria have been developed and/or established (17, 63–66). In this context, the Basque scientist Joan Trujols introduced the term “user generated” (versus “user-valued” and “-centered”) to elucidate not only the orientation of a scale but also its ways of generation (67). Referring to our research aim- the evaluation of users' experiences- we affirm that it is essential for researchers with experiential expertise to be included in all steps of a research process and to be entitled to have substantial decision-making power.

Contrasting to this assertion, and as stated in the introduction, participatory, user or collaboratively generated scales are still scarce. Our research tool shares features with the VOICE instrument that is designed to evaluate experiences and opinions on psychiatric treatment (12). Although the items in VOICE are aimed more at evaluating the structural quality of the provision of mental health care, similar items can be found in both scales, for example the question of continuity of everyday activities or the high level of availability of support from staff. In contrast, the CEO-MHS Questionnaire designed by Oades et al. and equally based on a participatory construction process, is an instrument to measure satisfaction and therefore refers less directly to the situational and objectifiable experiences of psychiatric treatment (14). Finally, the items of the PREM construct by Wallang (68) resemble in their operationalization (I-sentences) and to some extent also domains (“I feel safe,” “I feel supported,” “I feel independent” etc.) but unfortunately lacks a clear description of how its development was co-produced.

In contrast, PREM mental health scales that have been developed in conventional, non-participatory ways widely diverge in their domains and operationalization from the research tool developed in our project: As much as we appreciate (69) stressing on the need of PREM scales for scientific or routine evaluation, the domains of their scale do not seem to sufficiently specify, what they mean by “quality” or “good care.” As answers to these questions can only be normative, a lack of participatory engagement in their developmental process seems to be perilous. Thomas et al. (70) developed a PREM scale for evaluating the experiences of an emergency department, and, thus, depart from our project in their research aim. The DIALOG instrument incorporates both PROM and PREM items, the latter being only a few and rather broad in their scope (71). These only few examples, as well as our attempt of comparison, underscore the urgent need for collaborating with researchers with experiential expertise in the construction of PREM scales. As stated by various authors (4, 71), user-oriented services may only develop if the instruments to evaluate them will be better grounded in their perspectives and experiences into the future.

### Limitations

The participants of the general study sample were recruited from very diverse mental health hospital departments and therefore may differ to those in the pilot study sample that was conducted in only one department, in which some of FIT related aspects, e.g., home treatment, were barely implemented. Further, the limited project resources did not allow for a broader participatory negotiation of the developed, experiential components beyond the expert panel and the qualitative part of our process evaluation. Maybe as a result, they focus on experiences and needs of a “higher order,” rather neglecting more basic aspects of the service delivery, such as spaces for privacy, the quality of the served food, or the hygienic conditions of the treatment context. Fourth, we relied on self-report measures for assessing needs and experiences in psychiatric treatment which may have resulted in both error and bias in their measurement. As stated above, objective measurements of needs and experiences were not used and wait for further development. Our lack of a “gold standard” metric against which to compare needs and experiences limits our understanding of their concurrent validity. Finally, this study had a cross-sectional design: additional longitudinal studies in different mental health care settings are needed to establish psychometric properties of the NEPT research tool over time.

### Conclusions

Our project resulted in a psychometrically robust, object-appropriate, preliminary research tool that in its orientation corresponds to the interests and knowledge of users and so-called survivors of psychiatric treatment. As such, it may be perceived as a contribution to better align mental health care with the inherently value-based experiences and judgments of their users, an endeavor that is so urgently needed (4, 72, 73). The greatest methodological strength of our work is the systematic form of collaboration between researchers with and without experiential expertise within the framework of a prospective, controlled mental health services research design. By adopting a participatory process evaluation method, this collaboration took place in each study phase, which led to the results described above. Thus, although being constrained by the confines of a mental health service research epistemology, this collaborative knowledge production was possible at the level of process evaluation and can be reproduced accordingly in other projects.

We conclude by taking a critical look at the inevitable “side effects” of such an approach. There is great debate over the extent to which the provision of specific knowledge and approaches of survivors and researchers with experiential expertise in the context of projects such as ours are appropriated or co-opted by psychiatry without actually improving the conditions of care or services (74). Since the influential text of the American activist Judi Chamberlin ”On our Own“ (75), the question remains as to whether the experiential knowledge of people and researchers with experiential expertise are more useful if primarily incorporated into the conceptual and practical development of alternatives to psychiatry. We, as authors, are not sure how to answer this question, but it is important for us to point out the danger of such appropriation, also to ensure a continuous and fundamental problematization of this topic in similar projects of participatory and collaborative research.

## Data Availability Statement

The original contributions presented in the study are included in the article/Supplementary Material, further inquiries can be directed to the corresponding author/s.

## Ethics Statement

The studies involving human participants were reviewed and approved by an Ethics Vote of the TU Dresden dated 07.09.2017 was available. The patients/participants provided their written informed consent to participate in this study.

## Author Contributions

SP and HK were responsible for the draft of this manuscript. RKG, JZ, LG, PJ, TB, and YI contributed to the research process, interactive reviewing, literature search, interpretation of literature, and helped to draft the final version of the manuscript. CS, AN, FB, BS, MH, and JS revised the article critically. All authors approved the final version to be published.

## Funding

We acknowledge funding by the MHB Open Access Publication Fund supported by the German Research Association (DFG). The Psych Care study was financed by the German Innovation Fund of the Federal Joint Committee G-BA (No. 01VSF16053).

## Conflict of Interest

The authors declare that the research was conducted in the absence of any commercial or financial relationships that could be construed as a potential conflict of interest.

## Publisher's Note

All claims expressed in this article are solely those of the authors and do not necessarily represent those of their affiliated organizations, or those of the publisher, the editors and the reviewers. Any product that may be evaluated in this article, or claim that may be made by its manufacturer, is not guaranteed or endorsed by the publisher.
