# Supplementary material for: Needs and Experiences in Psychiatric Treatment (NEPT)- Piloting a Collaboratively Generated, Initial Research Tool to Evaluate Cross-Sectoral Mental Health Services
Source: Front Psychiatry. 2022 Jan 27;13:781726. doi: 10.3389/fpsyt.2022.781726 (PMC8829038; doi:10.3389/fpsyt.2022.781726)
Supplement: Supplementary file 1 [file Data_Sheet_1.pdf]

Annex:

The following constructs and the corresponding questions were developed jointly by people with and without their own psychiatric or recovery experience. The development took place in the course of the last year within the framework of a health care research project (PsychCare), which examines the effects of innovative care models in hospital psychiatry (so-called model care according to §64b Social Code V). The project is funded by the Innovation Fund and uses a controlled design, i.e. the effects of this innovative model care according to §64b are compared with those of standard care.
A sub-project of PsychCare uses qualitative methods and is equipped with researchers with and without experience expertise. Within this sub-project, we are currently developing a questionnaire that all patients recruited from both model and standard care will have to fill out from April of this year. We want to investigate whether the model care according to §64b really has better effects than the standard care.
In order to create the questionnaire, we used existing features that characterise specific processes and structures of the care models according to §64 to develop new features that capture what is special about these care models from the patients' perspective. This required a multi-step development process. We have called this dimension quality of experience (versus process, structural and outcome quality) and thus want to contribute to the fact that in the future the concrete experience of patients will also be systematically recorded in health services research.
Below are the 12 characteristics. They are defined and each is accompanied by a core sentence which is deliberately in the first person in order to do justice to the experiential dimension. In formulating the characteristics, we use the term "treatment" because it is often used, although we find it problematic in many respects. By treatment we always mean full inpatient, day-care and outpatient treatment in a hospital. Following the English usage, we use the term "users" instead of "patients".
For each characteristic, there are 2-3 alternative questions that are supposed to capture the essence of this characteristic. We ask you to assess whether this is really the case, i.e. which of the questions mentioned capture the essence of the associated characteristic. Please use the numbers from 1 to 3, where 1 stands for "essential", 2 for "appropriate but not essential" and 3 for "not essential".
Attention: The last characteristic and the corresponding questions may refer to stressful experiences. Please decide for yourself whether you want to answer these questions.
Thank you for your support and time!

Table A1

Nr.	**Component**	**Definition/core sentence**	**Question**	**Assessment**
1	Flexibility	Definition: A person experiences that they can choose the treatment setting and adapt it flexibly to their own needs.<br><br>Core sentence: I have different options.	Overall, I am treated in the settings that suit me best (full inpatient, day clinic, at home).	
			Changes run between different settings in such a way that it was suitable for me.	
2	Activity	Definition: A person experiences him/herself as active and	I am supported in developing activities	

		effective through the treatment. Activity can refer to concrete activities, but also to "inner" processes, such as thinking, making decisions, etc.<br><br>Core Sentence: I move.	that are helpful for me (activity can refer to concrete activities, but also to "inner" processes such as thinking, making decisions, etc.).	
			Through the treatment, I become more active overall (activity can refer to concrete activities, but also to "inner" processes, such as thinking, making decisions, etc.).	
			The treatment conditions lead me to become active (activity can refer to concrete activities, but also to "inner" processes, such as thinking, making decisions, etc.).	
3	(Avoidance of) stigmatisation	Definition: a) A person experiences him/herself in the treatment as correct, desired and in relationship with him/herself (avoidance of self-stigmatisation). b) A person is left alone and accepted by the staff as he or she is (avoidance of stigmatisation by others).<br><br>Core sentence: a) I am not wrong/ don't need to change/ be normal (self-stigmatisation) / b) You are not wrong/ don't need to change/ be normal (other-stigmatisation).	Overall, I feel "taken in" by the staff.	
			I feel taken seriously and accepted by the staff as a whole.	
			The treatment conditions (behaviour of staff, premises, regulations) allow me to look at myself favourably.	
4	Compatibility to everyday life	Definition: A person experiences that they can maintain or continue or develop their everyday life during or despite treatment.<br><br>Core sentence: I am not being pulled out.	I am able to maintain everyday activities (e.g. family and friends, hobbies, errands) during treatment.	
			I am supported during my treatment to develop skills that I can also use in my life.	
5	Autonomy	Definition: A person experiences themselves as self-determined and responsible in the treatment; they is supported in this by the	Overall, I have sufficient influence on decisions that are important to me during	

		staff, even if they may not always agree with all decisions.<br><br>Core sentence: I decide for myself and am supported in this.	treatment (e.g. regarding setting, medication, therapies).	
			I am supported in my decisions and decision-making during treatment.	
6	Safety	Definition: A person experiences reliable availability of support during treatment.<br><br>Core sentence: I am not alone.	I can rely on staff being there for me when I need help.	
			I experience support and security during the treatment.	
7	Continuity	Definition: A person experiences continuity. By continuity we mean that persons, processes and places have remained the same during the time of treatment.<br><br>Core sentence: I don't always have to start from the beginning.	I am mainly treated by the same staff members during the treatment.	
			During the treatment I experience continuity overall. (By continuity we mean that people, processes and places have remained the same during the time of treatment).	
			I don't have to start all over again during the treatment.	
8	Intensity	Definition: The circumstances of the treatment contribute to the person's ability to engage with the issues that concern them. This has changed the person's understanding of themselves and their situation.<br><br>Core sentence: The circumstances make it possible for me to engage with myself and for me to learn something for myself.	I am supported in the treatment to develop a clearer understanding of myself and my situation.	
			I am able to deal with my own situation during my treatment.	
9	Knowledge	Definition: A person experiences that their knowledge and competences are recognised and included during treatment. They receive the information they need to make informed decisions (e.g. about care services, hierarchies and other institutional conditions, etc.) in a barrier-free way and at the right time.	I am provided with information that is important for me (e.g. on procedures, regulations, treatments).	
			My knowledge about myself, my situation	

		Core sentence: My knowledge is used and information that is important for me is provided.	and my needs are sufficiently taken into account during the treatment.	
10	Time	Definition: A person experiences that he/she is given enough time/space/contact/conversation, e.g. to clarify his/her questions, to develop (at his/her own pace), to collect himself/herself, etc.<br><br>Core sentence: I get the time and space I need.	The time during treatment (talking time, contacts, free space etc.) is enough for me to change something for myself.	
			The time during the treatment is enough for me to change something for myself (e.g. I feel better, my problems are solved, etc.).	
			I am given enough time during the treatment.	
11	Solidarity/ Support	Definition: A person experiences treatment conditions that allow sufficient space for unmoderated exchange and mutual support between users.<br><br>Core sentence: Us (users) can stand by each other and have understanding for each other.	The exchange between users (joint activities, exchange of experiences, solidarity, etc.) is welcomed or even supported by the team.	
			The team encourages users to support each other.	
12	(Absence of) coercion and violence	Definition: A person does not experience coercion and violence during treatment.<br><br>Core sentence: Nothing will happen that I do not want.	I myself experienced coercive measures by the team during treatment (fixations, forced medication).	
			I myself have experienced other forms of coercion or duress during treatment, e.g. in relation to taking medication, participating in therapy, freedom of movement.	
			My impression is that attempts are made to prevent coercive measures during treatment.	
